# Guiding Hepatic Differentiation of Pluripotent Stem Cells Using 3D Microfluidic Co-Cultures with Human Hepatocytes

**DOI:** 10.3390/cells12151982

**Published:** 2023-08-01

**Authors:** Pouria Fattahi, Jose M. de Hoyos-Vega, Jong Hoon Choi, Caden D. Duffy, Alan M. Gonzalez-Suarez, Yuji Ishida, Kianna M. Nguyen, Kihak Gwon, Quinn P. Peterson, Takeshi Saito, Gulnaz Stybayeva, Alexander Revzin

**Affiliations:** 1 Department of Physiology and Biomedical Engineering, Mayo Clinic, Rochester, MN 55905, USA; fattahi@seas.upenn.edu (P.F.); dehoyos-vega.jose@mayo.edu (J.M.d.H.-V.); choi.jonghoon@mayo.edu (J.H.C.); cadenduf@gmail.com (C.D.D.); gonzalezsuarez.alan@mayo.edu (A.M.G.-S.); nguyen.kianna@mayo.edu (K.M.N.); khgwon85@gmail.com (K.G.); peterson.quinn@mayo.edu (Q.P.P.); stybayeva.gulnaz@mayo.edu (G.S.); 2 Department of Biomedical Engineering, University of Pennsylvania, Philadelphia, PA 19104, USA; 3 Department of Medicine, Division of Gastrointestinal and Liver Diseases, Keck School of Medicine, University of Southern California, Los Angeles, CA 90033, USA; yi_706@usc.edu (Y.I.); saitotak@usc.edu (T.S.); 4 Research and Development Unit, PhoenixBio Co., Ltd., Higashi-Hiroshima 739-0046, Japan

**Keywords:** microfluidics, co-culture, primary hepatocytes, embryonic stem cells, hepatic differentiation

## Abstract

Human pluripotent stem cells (hPSCs) are capable of unlimited proliferation and can undergo differentiation to give rise to cells and tissues of the three primary germ layers. While directing lineage selection of hPSCs has been an active area of research, improving the efficiency of differentiation remains an important objective. In this study, we describe a two-compartment microfluidic device for co-cultivation of adult human hepatocytes and stem cells. Both cell types were cultured in a 3D or spheroid format. Adult hepatocytes remained highly functional in the microfluidic device over the course of 4 weeks and served as a source of instructive paracrine cues to drive hepatic differentiation of stem cells cultured in the neighboring compartment. The differentiation of stem cells was more pronounced in microfluidic co-cultures compared to a standard hepatic differentiation protocol. In addition to improving stem cell differentiation outcomes, the microfluidic co-culture system described here may be used for parsing signals and mechanisms controlling hepatic cell fate.

## 1. Introduction

Human pluripotent stem cells (hPSCs) can proliferate indefinitely and give rise to cells of the three primary germ layers—endoderm, mesoderm, and ectoderm [1,2]. The potential of differentiating stem cells into any adult cell type makes them incredibly promising for cell-based therapies [3,4,5]. Most differentiation protocols deliver inductive cues (often growth factors) sequentially over the course of multiple days [6,7,8]. For example, a protocol for generating hepatocyte-like cells from hPSCs consists of three stages: (1) induction of endoderm with Activin A, (2) hepatic progenitor stage, and (3) hepatic maturation stage driven by hepatocyte growth factor (HGF) [9,10,11,12,13,14]. There have been a number of recent reports on the benefits of 3D (organoid/spheroid) cultures for hepatic differentiation and maturation of hPSCs [15,16,17,18,19]. Therefore, we wanted to implement a 3D culture format in this study.

Co-cultures, or the use of feeder layers, represent an important strategy for maintaining the pluripotent state of hPSCs. However, co-cultures may also be used to direct the differentiation of stem cells. Previous reports have shown that hepatic differentiation may be enhanced by co-cultivation with fibroblasts [20], liver stromal cells [21,22], and endothelial cells [23]. Our lab has previously described the use of micropatterned surfaces containing stem cells and adult hepatic or stromal cells in distinct but proximal locations and has shown that the presence of adult cells improved the differentiation of stem cells [24,25]. However, these previous examples of co-cultures were carried out in standard (large volume) cultures. We hypothesized that placing stem cells and adult cells inside microfluidic cultures may improve differentiation by increasing local concentrations of paracrine instructive signals.

Microfluidic devices utilize minimal amounts of media and may be automated to control the delivery of inductive cues and exchange of media, so as to minimize human involvement and chances of contamination [26]. Another, less appreciated benefit of microfluidic cultures has to do with accumulation of secreted signals under a diffusion-dominant culture regime [27,28,29,30]. Several reports have pointed to enhanced autocrine and paracrine signaling under such culture conditions [31,32,33,34]. Our team has shown that mouse PSCs maintained pluripotency in a microfluidic device by upregulating the production and signaling of endogenous leukemia inhibitor factor (LIF) [35]. We also demonstrated that adult hepatocytes exhibit enhanced phenotype and function in microfluidic devices by upregulating the production of hepato-inductive GFs, including HGF [36,37]. Additional studies by our team pointed to enhanced paracrine signaling for microfluidic co-cultures compared to large volume (transwell) co-cultures or the use of conditioned media [38,39].

In this study, we describe a microfluidic co-culture device comprised of two culture compartments interconnected by grooves to allow the exchange of paracrine signals. Each microfluidic compartment contained arrays of microwells to organize cells into spheroids. We demonstrated that human hepatocytes maintained high levels of hepatic phenotype and function over the course of 4 weeks in this microfluidic device. Stem cells seeded into the adjacent microfluidic compartment received instructive cues from adult cells and differentiated along the hepatic lineages. Hepatic differentiation in microfluidic co-cultures was more pronounced than in microfluidic monocultures or in a standard culture dish, which is attributed to the high local concentration of HGF and other signals secreted by adult hepatocytes.

## 2. Materials and Methods

### 2.1. Microfluidic Co-Culture Device Fabrication

#### 2.1.1. Fabrication of Microfluidic Co-Culture Devices

The microfluidic co-culture device consisted of the flow layer (top) for transport of media and the microwell layer (bottom) to organize 3D cell spheroids. The fabrication of the master molds for the microfluidic flow and microwell layers was carried using standard photolithography techniques and is described in Figure S1.

#### 2.1.2. Mold Fabrications

The flow (culture chamber) and microwells (bottom part of the device) master molds were fabricated using standard photolithography. Three photomasks with microfluidic patterns were designed using CAD software (version 2019, Autodesk Inc., San Francisco, CA, USA). The flow master mold was composed of two SU-8 resist layers to achieve microchannels 10 µm in height and a culture chamber 75 µm in height. First, a pristine 4-inch silicon wafer (452, University Wafer MS) was coated with SU-8 2010 (MicroChem, Westborough, MA, USA) and exposed to create the microchannel layer. After the post-exposure bake, a second 75 µm layer of SU-8 2025 (MicroChem, Westborough, MA, USA) was deposited to expose the flow layer. The microwell master mold comprised 84 microwells, each with a 300 µm diameter and 300 µm depth. For the later mold, a second pristine wafer was coated with SU-8 2100 (MicroChem, Westborough, MA, USA) to create a 300 µm layer. A mask aligner (KLOE mask aligner (KUB-3)) was used to UV-expose all SU-8 layers. All fabrication steps (spin coating, soft bake, exposure, post-exposure bake, development, and hard bake) were conducted according to the manufacturer’s instructions.

#### 2.1.3. PDMS Device Fabrication

Soft polymer devices were cast using the master molds under standard PDMS procedures. A 10:1 ratio of PDMS and curing agent (Ellsworth adhesive, Minneapolis, MN, USA) was poured over a silicon master, and a replica was molded by standard soft lithography procedures (Figure S1). Inlets and outlets from the cured flow layer (top) were punched using a 5 mm punch. Afterwards, the flow and microwell (bottom) PDMS layers were exposed to oxygen plasma for 2 min at 30 W (G-500 plasma cleaning system, Yield Engineering Systems, Livermore, CA, USA), and manually aligned under a stereoscope (Zeiss Stemi 508, Oberkochen, Germany). Four PYREX cloning cylinders (10 mm (d) × 10 mm (h)) were secured at the inlets and outlets using PDMS prepolymer and used as media reservoirs in static conditions. The aligned PDMS chips were then placed in an incubator overnight at 80 °C to strengthen the bond between the two layers and the cloning cylinders.

### 2.2. Cultures of Human Hepatocytes Isolated from the Liver of Humanized Liver Chimeric Mice

Chimeric mice with humanized livers (PXB-mice^®^) were generated using a previously reported method [40,41]. The cells were isolated from the liver of PXB-mice with a replacement index greater than 90% in the Saito lab at USC [42], plated, and cultured in T-75 flasks for 7 days to allow freshly isolated human hepatocytes for cell fate restoration, as described previously [43], followed by shipping to Mayo Clinic Rochester. The total time for hepatocyte cultures in a T-75 flask was 1 week. Cells were detached from the flask by treatment with 0.05% trypsin–EDTA for 15 min. The cells were then resuspended in 2% dimethyl sulfoxide-supplemented hepatocyte clonal growth medium (dHCGM) (200 μL with 8 × 10^6^ cells/mL) and seeded into a microfluidic device. Prior to cell seeding, microfluidic devices were made low-binding by overnight treatment with 1% Pluronic F-127 (non-ionic surfactant) in 1X PBS. After treatment, devices were rinsed with copious amounts of 1X PBS to remove excess Pluronic.

To achieve uniformity of cell seeding, 200 μL of cell suspension at a concentration of 8 × 10^6^ cells/mL was loaded into one of the media reservoirs (inlet) while keeping the outlet empty. The hydrostatic pressure between the inlet and outlet reservoirs drove cells into the device and filled the wells. After 2 min, we carefully collected cell suspension from both inlet and outlet reservoirs and added it to the opposite reservoir to populate wells again. This process was repeated six times to ensure uniform loading of cells into microwells (~84 spheroids or up to 1500 cells/each well). Excess cells were carefully removed by aspirating media, and 200 μL of dHCGM media was added into each reservoir. It is important to note that, during the whole process, the adjacent compartment was filled with 200 μL of media to avoid cross-contamination. The media were changed carefully every other day. The dHCGM contained DMEM supplemented with 10% FBS, 20 mmol/L HEPES, 15 µg/mL L-proline, 0.25 µg/mL insulin, 50 nmol/L dexamethasone, 44 mmol/L NaHCO3, 5 ng/mL EGF, 0.1 mmol/L ascorbic acid 2-phosphate, 100 IU/mL penicillin G, 100 µg/mL streptomycin, and 2% DMSO.

Cell viability was assessed in microfluidic devices. The devices were washed twice with 1X PBS to remove all media. The live/dead staining solution was prepared by adding 20 µL of ethidium homodimer and 5 µL of Calcein-AM (Live/Dead Viability/Cytotoxicity Kit, Thermo Fisher Scientific, Waltham, MA, USA) to 10 mL PBS. Then, 200 µL of live/dead solution was added to each device and incubated for 30 min at room temperature, followed by washing the cells using 1X PBS. An image was acquired in brightfield and fluorescence channels for Calcein-AM (ex/em 494/517 nm) and ethidium homodimer (ex/em 528/617 nm), using an inverted microscope (IX-83, Olympus, Tokyo, Japan).

### 2.3. Hepatic Differentiation of hPSCs under Standard Culture Conditions

The hPSCs, H9 SOX17-mCHERRY, were expanded on growth-arrested Mouse Embryonic Fibroblast (MEF) feeder layer. H9 cells were cultured in DMEM/F12 medium (Invitrogen, Carlsbad, CA, USA) with 20% knockout serum replacement (KSR), 1 mM L-glutamine, 1% nonessential amino acids, 0.1 mM β-mercaptoethanol, and 4 ng/mL basic fibroblast growth factor (Invitrogen). Induction of definitive endoderm (DE) was initiated using serum-free RPMI 1640 medium (Invitrogen, Carlsbad, CA, USA) supplemented with 100 ng/mL of Activin A (R&D Systems Inc., Minneapolis, MN, USA), 50 ng/mL Wnt3a, 2 mM L-glutamine, and 1% penicillin/streptomycin for 48 h. Then, the same medium without Wnt3a was supplemented with 1 × B27 supplement (Invitrogen) and 0.5 mM sodium butyrate for another 4–6 days. The DE cells were then detached with trypsin–EDTA and reseeded at a ratio of 2:1 on collagen I-coated 12-well plates (or microfluidic co-culture devices) for hepatic differentiation. The hepatic progenitor stage was induced with S2 media containing IMDM, +L-glu, +HEPES culture medium supplemented with 20% FBS, 1% Pen/Strep 100x, dexamethasone (100 nM), insulin (0.126 U/mL), 1-thioglycerol (0.3 mM), FGF-4 (20 ng/mL), HGF (20 ng/mL), BMP2, and BMP4 (10 ng/mL each) (R & D Systems). The day after seeding, old media were aspirated and replaced with fresh S2 media supplemented with 0.5% dimethylsulfoxide (DMSO). Cells remained in this medium for 9 to 14 days. Then, the cells were further differentiated and maintained in S3 culture medium (L15 medium) supplemented with 5% FBS, 1% Pen/Strep 100x, dexamethasone (100 nM), insulin (0.126 U/mL), FGF-4 (20 ng/mL), HGF (20 ng/mL), Oncostatin M (50 ng/mL) (R & D Systems), and 0.5% DMSO for 16 days. The S3 medium was changed daily during this stage.

### 2.4. Hepatic Differentiation of hPSCs in Microfluidic 3D Co-Cultures

HESCs were differentiated toward DE stage as described above, then collected from 12-well plates and seeded into a microfluidic device. At this point, a device containing adult hepatocyte spheroids in one compartment had DE cells seeded into the adjacent compartment. As mentioned above, the adult cell compartment was filled with 200 μL of dHCGM to prevent stem cells from crossing over. Then, we pipetted 200 μL of stem cell suspension with ~5 × 10^6^ cells/mL into the inlet of the stem cell compartment while keeping the outlet empty. This initiated flow into the cell culture compartment and filled the wells with cell suspension. The flow stopped within ~10 s as the level of liquid equilibrated between media reservoirs. At this point, cells settled down into the wells. We waited for 2 min before aspirating cell suspension and placing it in the opposite inlet (reservoir) to repeat seeding. We found this seeding method to help with uniform filling of wells with cells. The process was repeated four to six times to populate wells with sufficient density (~500–1000 cells per well). We then carefully removed the media by aspiration and added 200 μL of dHCGM media mixed with S2 media with only 25% inductive growth factors. The media were replaced with fresh media every other day.

### 2.5. Flow Cytometry

Stem cells were collected from standard multi-well plates for flow cytometry analysis at various timepoints throughout the 3-step differentiation. Cells were dispersed into single cells through incubation in TrypLE Express at 37 °C for 5 min, followed by fixation in 4% PFA at 4 °C. Cells were stored in PBS at 4 °C until staining. To stain the cells, the block solution (PBS + 0.1% Triton X-100 + 5% donkey serum) was added to the cells and incubated at room temperature for 40 min. Cells were incubated with primary and secondary antibodies at room temperature for 1 h each; dilution factors were performed according to the manufacturer’s recommendation. The antibodies are listed in the supplementary table (Table S1). The cells were washed three times with PBST, and the supernatant was aspirated. The cell suspension was treated with 100 μL of PBST before being analyzed with the Accuri C6 or Attune flow cytometers. FlowJo v10 software was used to analyze and process flow cytometry data.

### 2.6. Immunofluorescent Staining of Cells

Immunofluorescence staining was carried out according to a previously published method [44]. In brief, cell monolayers or spheroids were fixed in 4% paraformaldehyde (Electron Microscopy Sciences, Hatfield, PA, USA) for 15 min and incubated with a permeabilization buffer containing 2% bovine serum albumin (Sigma Aldrich, St Louis, MO, USA) and Triton X at 0.1% (Invitrogen) in phosphate-buffered saline (PBS) for 45 min at room temperature. Using the following primary antibodies, cells were stained for OCT4, SOX17, albumin, AFP, HNF1β, HNF4α, and human specific MRP2 and incubated overnight at 4 °C. Cells were incubated with secondary antibodies for 3 h at 4 °C. Excess secondary antibody solution was removed by flushing three times with fresh 1X PBS. Then, Alexa 488-labeled phalloidin (Invitrogen; 1:500, 30 min) and DAPI (1:5000, 5 min) were used to stain actin filaments and cell nuclei, respectively. Immunostaining samples were mounted with Fluoromount-G (SouthernBiotech, Birmingham, AL, USA). Afterwards, micrographs were acquired using a fluorescence microscope (Olympus IX83) and a confocal microscope (LSM 780, Zeiss Microscopy, Oberkochen, Germany) with ×10, ×20, and ×40 objectives.

### 2.7. ELISA

Albumin secretion was assessed using a commercial ELISA assay (Bethyl Laboratories, Montgomery, TX, USA). Albumin values were normalized to the cell number (84 spheroids or up to 1500 cells/each well). Hepatocytes were cultured in a microfluidic co-culture device as 3D and monoculture (2D microfluidic) for 33 days with daily media exchange. The media were collected every other day, and the albumin level secreted by hepatocytes was measured by ELISA. HGF, urea, and bile acid assays were carried out using HGF DuoSet ELISA (DY294) from R&D Systems (Minneapolis, MN, USA), QuantiChrom™ Urea Assay Kit (DIUR-100) from BioAssay Systems (Hayward, CA, USA), and Total Bile Acid Assay Kit (MET-5005) from Cell Biolabs (San Diego, CA, USA). All assays were performed per the manufacturers’ instructions. Three samples from hepatocytes’ conditioned media, from days 3, 7, and 14, were analyzed using a spectrophotometer (Synergy H1, BioTek Instruments, Winooski, VT, USA). *Secretome profiling*: Supernatants were collected from human hepatocyte spheroids monocultured in microfluidic devices and frozen at −80 °C. Secretome profiling analyses were conducted using Human Angiogenesis and Growth Factor 17-Plex and Human Cytokine/Chemokine 96-Plex discovery assays at Eve Technologies (Calgary, AB, Canada). Media from three devices (*n = 3*) were evaluated for days 3, 7, and 14.

### 2.8. RT-PCR Analysis of Hepatic and Stem Cell Gene Expression

Cells were collected by separating the two layers of the microfluidic device and collecting the cell spheroids from each chamber. For PCR analysis, total RNA was isolated using the High Pure RNA Isolation Kit (Roche Diagnostics, Mannheim, Germany), according to the manufacturer’s instructions. RNA was converted to cDNA using the Transcriptor First Strand cDNA Synthesis Kit (Roche), and the quantitative PCR reactions were set up using the FastStart Universal SYBR Green Master (Roche). All PCR reactions were performed in duplicate. Primers are listed in Table S2. The relative expression level of each gene was calculated using the comparative threshold cycle (ΔCt) method, with GAPDH as a housekeeping gene.

### 2.9. Modeling HGF Secretion and Distribution in the Microfluidic Device

A 3D model of the microfluidic device was drafted in AutoCAD and exported to COMSOL (v6.0, Burlington, MA, USA). The 3D model reflected the geometry of the microfluidic device and included hepatic spheroids (individual diameter = 171.4 µm). The HGF secretion rates were determined from experimental data (ELISA): 2.52, 2.84, and 3.54 × 10^−3^ ng‧spheroid^−1^‧h^−1^ at days 3, 7, and 14, respectively. The COMSOL model, including the HGF diffusion coefficient, was taken to be 8.5 × 10^−11^ m^2^‧s^−1^ [37] with the assumption of no flow in the device. The model generated HGF concentrations in 6 min increments over the course of 48 h.

### 2.10. Statistical Analysis

A minimum of three biological replicates were used for each condition. The data are represented as mean ± standard deviation (SD). Statistical significance between experimental groups was assessed using a two-tailed unpaired t-test. Differences were considered statistically significant at *p*-values < 0.05, with the value denoted by the following notation: * *p* ≤ 0.05, ** *p* ≤ 0.01, *** *p* ≤ 0.001, **** *p* ≤ 0.0001. GraphPad Prism (ver. 9; GraphPad Software) was used to statistically analyze the data.

## 3. Results

### 3.1. Design of a Microfluidic Device for 3D Co-Cultures of Hepatocytes and Stem Cells

**Figure 1:**
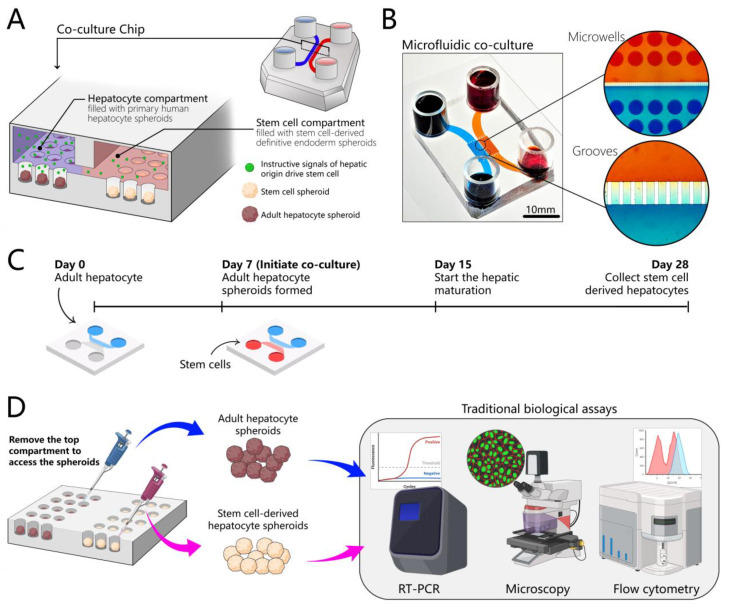
Design of the microfluidic 3D co-culture device. (**A**) The concept of the co-culture device: adjacent microfluidic compartments are populated with adult hepatocytes and stem cells. Both cell types are cultured as spheroids. Each compartment contains an array of wells designed to organize cells into spheroids. Compartments are interconnected by grooves to allow cross-talk via paracrine signals. Adult hepatocytes secrete instructive signals that drive the hepatic differentiation of stem cells residing in the neighboring compartment. (**B**) The images show the top view of an image of a microfluidic co-culture device. High-magnification images of grooves and microwells show the platform for communication between two groups of adult hepatocytes and stem cell spheroids through endogenous signals. (**C**) A multi-stage differentiation protocol in a co-culture device. (**D**) The design of the device allows for the collection of each cell type separately for phenotype and gene expression analysis. We can remove the top chamber, access the spheroids separately, and collect them, which makes our chip compatible with traditional biological assays such as immunofluorescence microscopy, RT-PCR, and flow cytometry.

Microfluidic devices were fabricated from silicone rubber (PDMS) using standard soft lithography techniques (Figures S1 and S2). The co-cultivation microfluidic device consisted of two layers: microwell layer (two sets of 84 wells, each well 300 µm in diameter and depth) (Figure 1A,B) and a flow channel layer (~75 µm tall). The device was made low-binding by treatment with Pluronic such that, upon seeding and landing in wells, cells organized into spheroids within 24 to 48 h. The two compartments were connected by grooves, allowing the exchange of paracrine signals (Figure 1A). Our microfluidic cultures did not have active flow or pumping, and were specifically designed to harness and locally accumulate signals secreted by adult hepatocytes for differentiation of stem cells.

Why use adult hepatocytes as the source of instructive cues for driving the hepatic differentiation of stem cells? Our team has previously demonstrated that adult rodent hepatocytes and liver explants cultured in microfluidic devices in the absence of flow upregulated the production of inductive cues (e.g., HGF, epidermal growth factor (EGF), insulin-like growth factor (IGF)-1) [37,45,46]. In fact, the phenotype of hepatocytes was dependent on the geometry of the culture chamber—taller chambers resulted in dilution of endogenous signals and a lower hepatic phenotype [34]. In this paper, we wanted to test the hypothesis that HGF and other signals produced by adult hepatocytes may be harnessed to drive the hepatic differentiation of stem cells. We wanted to establish syngeneic cultures and first focused on microfluidic cultures of adult human hepatocytes.

Human hepatocytes were isolated from the livers of humanized liver chimeric mice. This is a urokinase plasminogen activator (uPA)-severe combined immunodeficiency (SCID) mouse model where transplanted hepatocytes have a competitive advantage over transgenic host hepatocytes, to the extent that the hepatic parenchyma in these mice consist largely (~96%) of human hepatocytes [41,47]. Unlike cadaveric human hepatocytes, which are of variable quality, human hepatocytes are isolated from chimeric mice in a manner similar to rodent hepatocytes and are of reproducibly high quality.

These cells were seeded first and cultured in the device for 5 to 7 days, to give the cells time to acclimatize and to re-establish their hepatic phenotype in the microfluidic device. hPSCs were differentiated into definitive endoderm (DE) in standard cultureware and then seeded into the device that already contained hepatocytes. The co-cultures of human hepatocytes and DE stem cells were maintained for an additional three weeks (Figure 1C). Using co-cultures enables cell maturation in a way that is not routinely possible in 2D cultures, and, at the same time, it is easy to access spheroids and collect samples to test various biological assays (Figure 1D).

### 3.2. Long-Term Maintenance of Functional Human Hepatocytes

**Figure 2:**
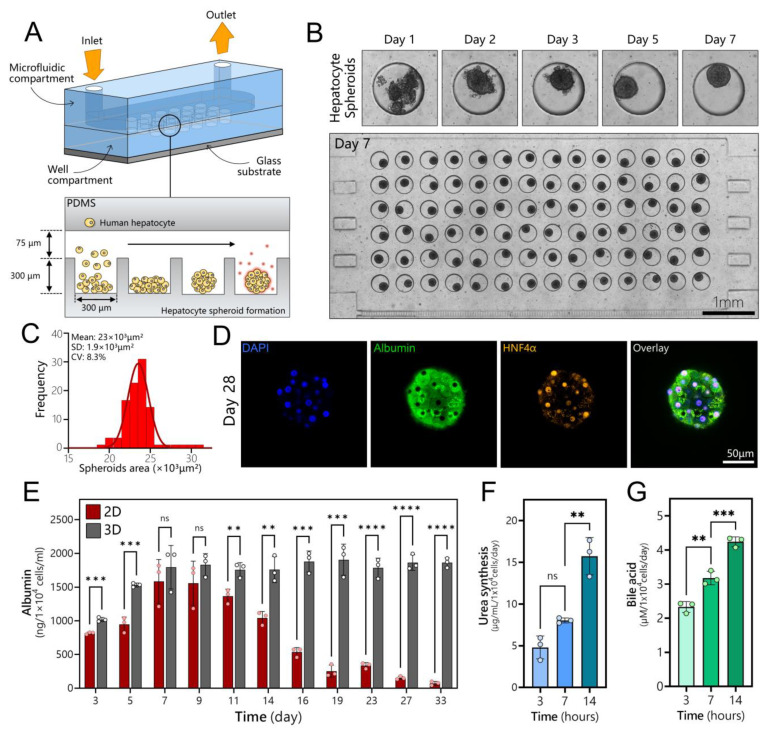
Long-term cultures of human hepatocyte spheroids in a microfluidic device. (**A**) Schematic describing seeding of cells and spheroid formation in a microfluidic device. Devices were made low-binding by treatment with Pluronic. This promoted cell aggregation into spheroids inside microwells. (**B**) A time-lapse sequence showing the aggregation of cells into a spheroid followed by spheroid compaction. An array of 84 hepatocyte spheroids is shown in the bottom image. (**C**) Spheroids were uniformly sized, with an average diameter of 171.4 ± 7.1 μm. (**D**) Immunofluorescence staining of a hepatocyte spheroid for hepatic markers (albumin and HNF4α) after 4 weeks of culture in a microfluidic device. (**E**) Production of albumin in a microfluidic monolayer (2D) and spheroid (3D) cultures of hepatocytes (*n* = 3). (**F**) Urea synthesis analysis for hepatocyte spheroid at 3, 7, and 14 days. (**G**) Bile acid production for hepatocyte spheroid at 3, 7, and 14 days. Data are shown as mean ± SD, ** *p* ≤ 0.01, *** *p* ≤ 0.001, **** *p* ≤ 0.0001, ns (not significant).

Human hepatocytes were seeded into a compartment of the microfluidic co-culture device (Figure 2A). The two compartments were identical in dimensions and had 84 wells each. The cells began to aggregate 24 h post-seeding, formed spheroids after 2 days, and appeared to undergo compaction/condensation until day 7, stabilizing at a diameter of 171.4 μm with a coefficient of variation (CV) of 4.1%. Figure 2B shows an array of uniformly sized hepatocyte spheroids in a compartment of a microfluidic device, while Figure 2C provides a histogram of spheroid size distribution. Importantly, hepatocyte spheroids remained highly functional and expressed markers of a well-differentiated phenotype after extensive cultivation. Figure 2D shows immunofluorescent staining for albumin, a hallmark indicator of liver function, and HNF4α, another important marker of the hepatic phenotype, after 28 days of culture. Both markers were strongly expressed in hepatocyte spheroid cultures at this late timepoint. We also examined albumin secretion, urea synthesis, and bile acid production from hepatocytes that were maintained in microfluidic spheroid cultures (Figure 2E–G), and we then compared the albumin secretion in microfluidic spheroid cultures to microfluidic 2D or monolayer cultures. These results, presented in Figure 2E, demonstrate that, while hepatocytes remained functional in 2D cultures for 14 days, their long-term function was dramatically enhanced in 3D cultures, where robust production of albumin extended to 33 days.

### 3.3. Polarization, Biliary Excretion, and Gene Expression in Microfluidic Spheroid Cultures of Adult Hepatocytes

**Figure 3:**
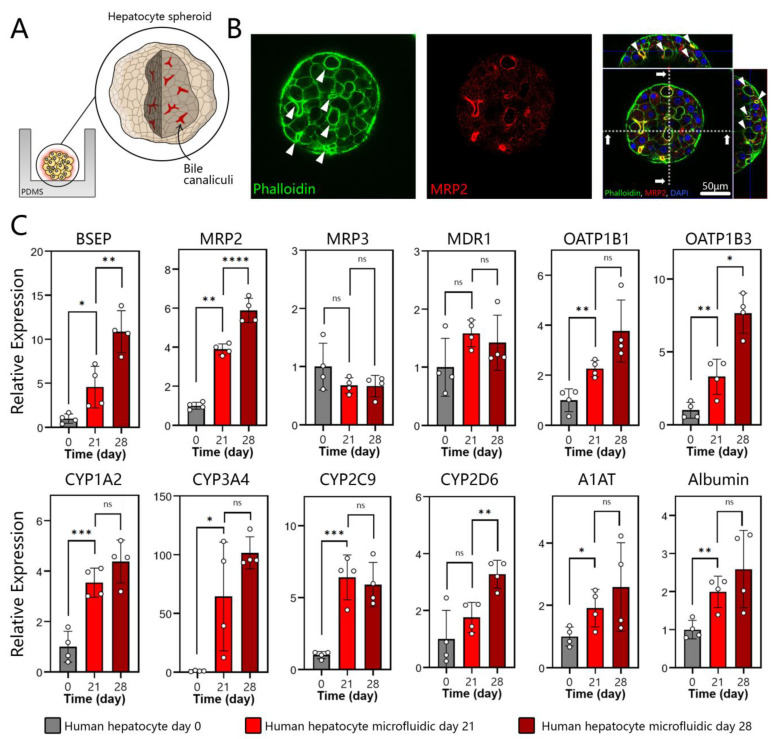
Polarization and hepatic gene expression in microfluidic spheroid cultures of human hepatocytes. (**A**) A cartoon describing the localization of bile canaliculi to the apical and lateral domains of hepatocytes within a spheroid. (**B**) Immunofluorescence imaged by confocal microscopy at day 28 of cultures. Hepatocyte spheroids were stained with phalloidin for F-actin (green) and MRP2 (red). A representative image shows that the bile acid transporter MRP-2 and actin were arranged along the cell periphery in an organized pericanalicular pattern. (**C**) RT-PCR analysis for markers of a well-differentiated hepatic phenotype: bile transporter proteins (BSEP, MRP2, MRP3, and MDR1), drug transporters (OATP1B1 and OATP1B3), CYP450 enzymes (CYP1A2, CYP3A4, CYP2C9, and CYP2D6), and serum proteins produced by the liver (A1AT and albumin). Data are represented as mean ± SD, * *p* ≤ 0.05, ** *p* ≤ 0.01, *** *p* ≤ 0.001, **** *p* ≤ 0.0001, ns (not significant) (*n* = 4 per group).

Unlike typical apical/basolateral polarization of columnar epithelium, a hepatocyte is polarized to contain protein transporters in the basal domain and bile acid transporters in the apical and lateral domains. Polarized and well-differentiated hepatocytes are expected to excrete bile into canaliculi that drain it into intrahepatic bile ducts [48]. MRP2 is a transporter responsible for the delivery of amphiphilic anionic conjugates (e.g., bilirubin glucuronides) into bile, and is expressed in well-differentiated hepatocytes [49]. As seen from immunofluorescence images in Figure 3A,B, MRP2 is expressed strongly in spheroids and is co-localized with actin. The regions with MRP2 localization (white arrows in Figure 3B) strongly suggest polarization of hepatocytes and the existence of bile canaliculi in the spheroids. A z-stack projection in Figure S3B helps to better appreciate the bile canalicular network inside a hepatocyte spheroid. Additional analysis of transporter gene expression was carried out by RT-PCR. As shown in Figure 3C, gene expression of BSEP and MRP2—transporters responsible for taurine and glutathione conjugates, respectively [50]—was enhanced after 28 days of culture, while gene expression for MRP3 and MDR1 remained the same. MRP3 is a type of transporter located on the basolateral membrane of the hepatocyte that secretes glucorinated drug metabolites into the bloodstream [51,52]. MDR1 is localized on the apical membrane of the hepatocytes and serves to transport hydrophobic drug metabolites into the bile canaliculi [51,53]. We note that, while the day 0 timepoint denotes hepatocytes at the time of seeding into microfluidic devices, these cells were collected from 2D cultures and, therefore, may also be thought of as a 2D standard culture control. It is therefore telling that, after 4 weeks of culture, bile transporter gene expression is either better than or similar to that of hepatocytes at the time of seeding.

In addition to bile acid production, the liver is responsible for metabolizing xenobiotics present in the circulation. These molecules are regulated via drug transporters, including the organic anion-transporting polypeptides 1B1 (OATP1B1) and 1B3 (OATP1B3) located in the sinusoidal (basal) side of a hepatocyte [54]. As seen from Figure 3C, genes for both OATP1B1 and OATP1B3 were expressed at the highest level at day 28, several fold higher than for 2D hepatocyte cultures at the time of seeding into a microfluidic device. Once taken up by the hepatocytes, xenobiotics (toxicants and drugs) are metabolized by a family of cytochrome P450 enzymes (CYPs). We assessed a panel of CYPs, including CYP1A2 (propranolol) [55], CYP3A4 (clarithromycin) [56], CYP2C9 (Ibuprofen) [57], and CYP2D6 (desipramine) [58]. Figure 3C shows that gene expression levels for these enzymes increased over time and were at their highest level on day 28 of culture. For all four CYP isoforms characterized, gene expression in microfluidic 3D cultures was manyfold higher compared to 2D hepatocytes at the time of seeding. The fold increase ranged from 4.4 for CYP1A2 to ~100 for CYP3A4.

The benefits of microfluidic cultures were also obvious when evaluating gene expression for albumin and α1-antitrypsin (A1AT), serum proteins produced by the liver. Gene expression for both proteins was at its highest level after 28 days of 3D cultures in a microfluidic device. Taken together, our data demonstrate that human hepatocytes cultured as spheroids in a microfluidic device exhibited polarity and gene expression consistent with a well-differentiated hepatic phenotype. We hypothesized that such microfluidic cultures may secrete instructive signals to promote the differentiation of neighboring stem cells. We focused on hepatocyte growth factor (HGF)—a key morphogen in liver development and regeneration processes.

### 3.4. Assessing Production of HGF and Other Secreted Signals in Microfluidic Hepatocyte Cultures

**Figure 4:**
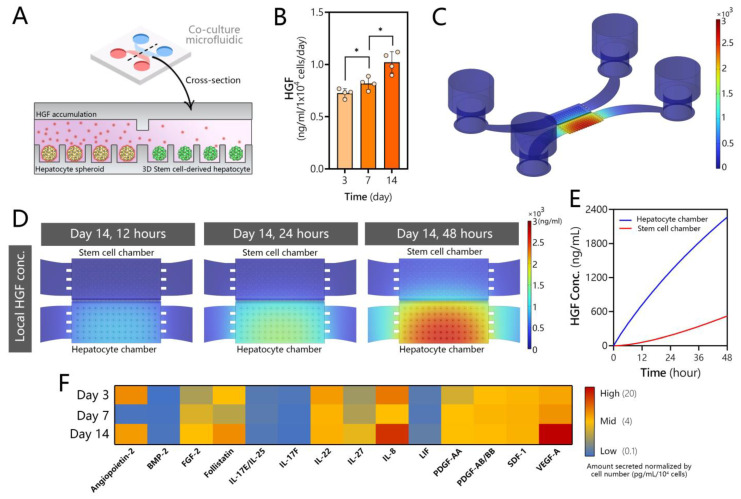
Secretome analysis for microfluidic human hepatocyte spheroid cultures. (**A**) Schematic of the microfluidic co-culture showing adult hepatocytes producing HGF and other signals that diffuse into the neighboring stem cell compartment. (**B**) HGF ELISA of media collected from microfluidic spheroid cultures of human hepatocytes. Data are represented as mean ± SD, * *p* ≤ 0.05 (*n* = 4 per group). (**C**) COMSOL modeling of HGF secretion, accumulation, and diffusion in the microfluidic device at t = 48 h. Note that the signal is localized to the hepatocyte compartment. (**D**) COMSOL modeling heatmaps of HGF accumulation in the hepatocyte and stem cell compartments over time. (**E**) Model-predicted increase of HGF concentration in hepatocyte and stem cell compartments over the course of 48 h. The graph reports the average concentrations of HGF in each culture compartment. (**F**) Signals secreted by human hepatocytes with known roles in hepatic differentiation, proliferation, and maturation.

HGF is the key signal driving early hepatic specification as well as maturation during liver development [59]. Because of its importance in development, HGF is a mainstay in directed hepatic differentiation protocols for hPSCs. Such protocols are often subdivided into DE, hepatic progenitor, and hepatic maturation stages, with HGF present at the maturation stage in concentrations ranging from 10 to 20 ng/mL [60]. It is also worth mentioning that HGF is unstable, with a half-life of <15 min [61], which likely limits its bioactivity when added as a bolus into media. We hypothesized that adult hepatocytes in the microfluidic co-culture system may produce HGF molecules in a continuous manner, in sufficiently high concentrations to affect differentiation of the stem cell compartment (see Figure 4A).

While GF production is typically attributed to nonparenchymal cells of the liver (e.g., stellate cells [62]), it has long been appreciated that hepatocytes express genes for multiple GFs relevant to the normal and fibrotic states of the liver [63,64]. Our team has shown that 2D and 3D cultures of rodent hepatocytes as well as explant liver cultures upregulate production of HGF in microfluidic devices under a diffusion-controlled regime (absence of flow) [37,45,46]. In fact, the hepatic function of these cultures was dependent in large part on HGF signaling and was degraded when this signaling was inhibited. In the present study, we characterized the production of HGF in human hepatocyte cultures. As may be seen from ELISA results in Figure 4B, microfluidic spheroid cultures of human hepatocytes produced HGF robustly (~1 ng/mL), with levels increasing over time in parallel with increased functionality of cells (see, for example, albumin synthesis data in Figure 2E). We note that ELISA measurement required the collection of all media from the microfluidic device—a process that mixed cell-conditioned media in the small-volume culture chamber (3 µL) with media from reservoirs (~200 µL).

In order to determine the local concentration of HGF in the culture chamber around hepatocyte spheroids, we set up a COMSOL model incorporating device geometry, cell number, and secretion rate based on ELISA measurements. The results of the modeling are presented in Figure 4C,D. They highlight that 96% of secreted HGF was expected to be present at the source, in the cell culture chamber, with <4% of HGF distributed across the transport channel and media reservoirs. Thus, the design of the microfluidic device enabled accumulation of this signal in a specific location within the device, in the vicinity of the stem cell compartment. Our modeling highlighted that HGF concentration built up rapidly over the course of 48 h (time between media exchanges), reaching 2 µg/mL in the source (hepatocyte) chamber. The model also calculated that the recipient (stem cell) compartment was exposed to a high concentration of HGF, reaching 600 ng/mL at the 48 h timepoint. While our model was simplified and did not take into account the internalization or degradation of HGF, the results are compelling as they suggest that stem cells are exposed to an order-of-magnitude higher level of HGF in our microfluidic co-culture system compared to standard hepatic differentiation. In addition, HGF and other inductive signals are produced continuously by adult cells in the microfluidic device and are likely more bioactive than in standard differentiation protocols.

What would happen if co-culture experiments were performed in large-volume cultures? Our microfluidic device is designed specifically to promote local accumulation of signals secreted by the adult hepatocytes next to stem cells. The design parameters that influence concentration gradients of signals are the number of cells in the culture chamber, the dimensions of transport channels leading from the culture chamber to the media reservoir, and the dimensions of the culture chamber itself. To demonstrate the importance of the geometry of the culture format, we modeled two hypothetical scenarios: (1) a scenario where the height of the culture chamber in the microfluidic co-culture device was not 75 µm as in the original design but 1 mm and (2) a scenario of hepatocyte spheroids and stem cells separated by a 1 mm column of media in a transwell configuration. For this modeling, the HGF secretion rate was assumed to be the same as in the original microfluidic device—likely an overestimation. Modeling results presented in Figure S4 suggest that HGF concentrations experienced by stem cells in both scenarios were at least 10-fold lower than in the original low-volume microfluidic device, ~43 ng/mL and ~57 ng/mL for scenarios 1 and 2, respectively. These results underscore the potential benefits of optimally designed microfluidic devices for harnessing secreted signals.

To further explore the signals through which adult hepatocytes may induce differentiation of DE cells, we conducted secretome analysis on conditioned media from microfluidic human hepatocyte spheroid cultures (Figure 4F). We found that hepatocytes release multiple growth factors, other than HGF, that are known to play a role in hepatoblast formation and proliferation, along with those that influence hepatocyte differentiation, proliferation, and maturation. For example, the conditioned media contained high levels of fibroblast growth factor 2 (FGF2), which has been reported to initiate hepatic gene expression to generate hepatoblasts and mark a split between pancreatic and hepatic lineages following DE stage [65]. Hepatocyte cultures also produced IL-17 and IL-22, which have been shown to play a role in proliferation of hepatoblasts [66,67], as well as IL-27, platelet derived growth factor (PDGF), leukemia inhibitory factor (LIF), and stromal cell derived factor 1 (SDF-1), all of which have been reported to play roles in inducing hepatic differentiation [67,68,69,70]. BMP-9 may play a role in hepatocyte maturation [71], and follistatin enhances hepatocyte proliferation [72]. IL-8, secreted by microfluidic hepatocyte cultures at high levels, has been shown to play a role in transdifferentiation of mature hepatocytes into cholangiocytes and, thus, could later be advantageous for generating complex multi-cellular organoids [73]. Of note, VEG-F and angiopoietin-2 were also present at high concentrations in media conditioned by hepatocytes. These GFs play a role in endothelial development but, given that these cells are already differentiated towards definitive endoderm, endothelial lineage is likely inaccessible in our system. On the whole, our analysis reveals that microfluidic hepatocyte cultures produce multiple signals, including but not limited to HGF, that have been implicated in various stages of hepatic differentiation. As noted at the beginning of this section, the ability of hepatocytes to produce cytokines and growth factors is generally underappreciated. Typical preparations of primary hepatocyte cultures have >90% purity but leave open the possibility that nonparenchymal cells are present and shape the secretome to a certain extent. We believe that isolating human hepatocytes from chimeric mice and using a human-specific immunoassay for secretome analysis makes this possibility unlikely.

### 3.5. Creating Co-Cultures to Guide Hepatic Differentiation of Stem Cells

**Figure 5:**
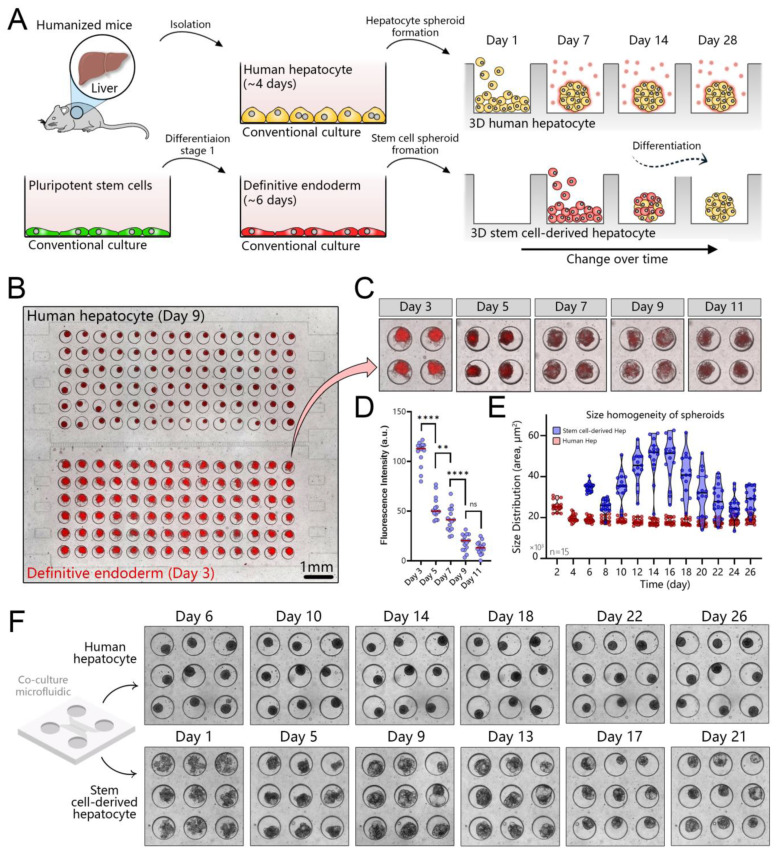
Establishing co-cultures of human hepatocyte and stem cell spheroids in a microfluidic device. (**A**) Workflow for establishing a co-culture. Human hepatocytes are isolated from mice with humanized livers, seeded into one compartment of the co-culture device, and cultured for 7 days to promote spheroid formation. Pluripotent stem cells are differentiated into definitive endoderm (DE) in a standard culture plate and then seeded into the device containing hepatocyte spheroids. (**B**) An image showing hepatocyte and DE stem cell spheroids in a co-culture device. Note that stem cells contained the mCherry (red fluorescence) reporter for SOX17—marker of DE. (**C**,**D**) The red fluorescence signal decreases as stem cells differentiate out of the DE stage and toward a hepatic fate. (**E**) Comparison of spheroid area for adult hepatocytes and stem cells. The latter undergo considerable changes in size in the process of differentiation. (**F**) Images showing hepatocyte and stem cell spheroids at different points during 4 weeks of culture, ** *p* ≤ 0.01, **** *p* ≤ 0.0001, ns (not significant).

The workflow of a co-culture experiment is shown in Figure 5A,B. hPSCs were first differentiated to definitive endoderm (DE) and then seeded into a microfluidic device already containing adult hepatocyte spheroids. Figure 5B shows an image of hepatocyte and DE stem cell spheroids residing in adjacent microfluidic compartments interconnected by microgrooves (500 µm in length and 10 µm in height). A reader may note that DE stem cells were fluorescent. These cells were genetically engineered to co-express a DE marker, SOX17, with mCherry [74].

The intensity of fluorescence decreases as stem cells differentiate out of the DE stage and along the hepatic lineage (Figure 5C,D). Stem cell spheroids changed in size during differentiation (Figure 5E). Similar observations of proliferation and cell death over the course of the differentiation process were made by us previously when working with spheroids in suspension cultures [44]. Conversely, we found that the size of adult hepatocyte spheroids remained unchanged during 28 days of microfluidics culture (Figure 5F).

### 3.6. Assessing Hepatic Differentiation of DE Stem Cells in Co-Cultures with Adult Hepatocytes

**Figure 6:**
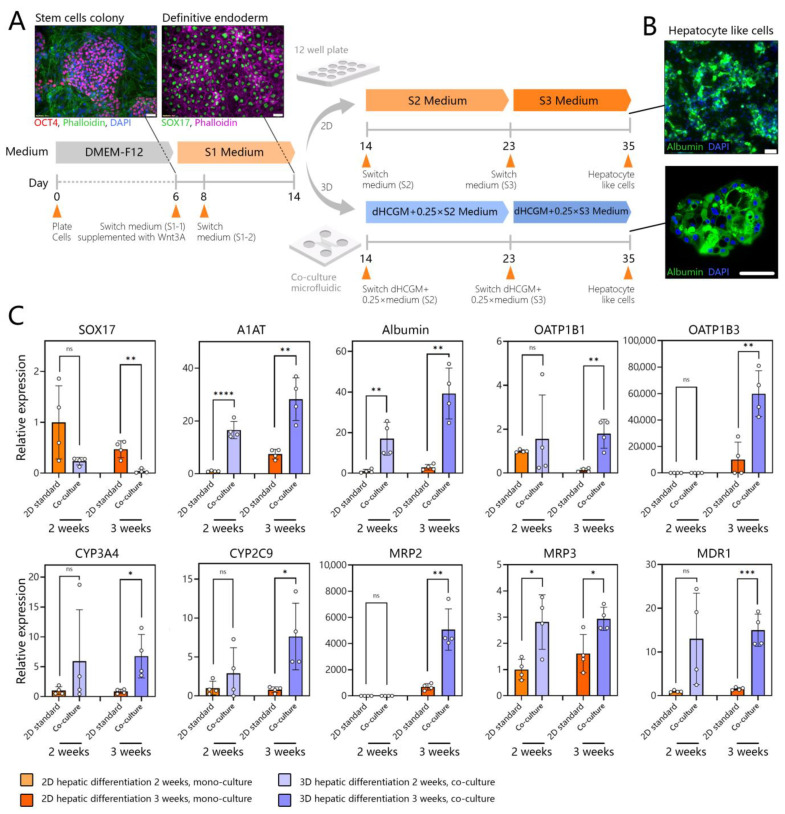
Assessing hepatic differentiation of stem cells in microfluidic 3D co-cultures. (**A**) Differentiating hPSCs (H9 cells) using standard and microfluidic protocols. S1 media promotes DE stage, S2—hepatic progenitor stage, S3—hepatic maturation stage. hPSCs are differentiated in standard cultures into DE stem cells and then placed into a microfluidic device that already contains adult hepatocytes. Subsequent differentiation in the microfluidic device is performed in combination media comprised of 3:1 ratio of hepatocyte maintenance:stem cell media. Microfluidic differentiation is benchmarked against a standard protocol carried out in multi-well plates. (**B**) Representative images showing albumin expression in SC-hepatocytes at the end of the standard and microfluidic differentiation protocols. (**C**) RT-PCR analysis of DE marker (SOX17), hepatic proteins (A1AT and albumin), drug transporters (OATP1B1 and OATP1B3), CYP450 enzymes (CYP3A4 and CYP2C9), and bile transporters (MRP2, MRP3, and MDR1). Data are presented as mean standard deviation, * *p* ≤ 0.05, ** *p* ≤ 0.01, *** *p* ≤ 0.001, **** *p* ≤ 0.0001, ns (not significant).

Next, we wanted to evaluate hepatic differentiation in microfluidic devices. Three experimental groups were set up: (1) microfluidic mono-cultures, (2) a standard differentiation protocol in 2D culture plates, and (3) microfluidic co-cultures adapted from Zern et al. [75]. The workflow for this set of experiments is shown in Figure 6A. hPSCs (H9 cells) were expanded on mouse embryonic fibroblasts (MEFs) for 8 days, as described previously [76]. Pluripotent stem cells were differentiated into stage 1 (DE) by exposure to activin A and Wnt3A for 48 h, followed by 6 days of DE2 media (no GFs). Immunofluorescence staining confirmed that stem cells lost expression of the pluripotency marker Oct4 and gained the DE marker SOX17 at the end of stage 1. After completion of stage 1, DE stem cells were collected and reseeded either into collagen-coated multi-well plates or into microfluidic devices. In multi-well plates, stem cells were differentiated into hepatic progenitors (stage 2) for 9 days and then exposed to hepatic maturation signals (stage 3) for 12 to 16 days. Key inductive cues for stages 2 and 3 were BMP4 and HGF, respectively. A detailed composition of the media may be found in the Methods section. In a standard multi-well plate, beginning with stage 2, stem cells exhibited polygonal morphology consisting of epithelial cells (Figure S5). RT-PCR and flow cytometry analyses (Figure S6) confirmed that our differentiation protocol produced results similar to those reported in the literature, with 93.8% SOX17+/OCT4− cells at the end of stage 1, 16.2% albumin+ cells at the end of stage 2, and 63.4% albumin+ cells by the end of stage 3 (Figure S5). It is worth noting that, during the differentiation process, cells underwent morphological changes and acquired cuboidal hepatic morphology at the end of stage 3.

DE stem cells were also collected from multi-well plates and transferred into a microfluidic device to create mono- or co-cultures. It has been reported that high initial plating density is important for the DE stage [77] and for final differentiation yield [78]. This is because a significant fraction of stem cells undergo apoptosis in the process of DE differentiation. Given that a microfluidic compartment housed a relatively small number of cells (84 spheroids, or up to 1500 cells/well) we found it beneficial to begin microfluidic differentiation at the DE stage and not the pluripotent stage. DE stem cells were seeded into microfluidic devices to create either mono- or co-cultures with adult hepatocytes. The co-cultures were exposed to mixed media comprised of dimethyl sulfoxide-supplemented hepatocyte clonal growth medium (dHCGM, or hepatocyte maintenance media) and S2 or S3 media (Table S3) for stem cells. This reduced the reliance on media supplemented with inductive growth factors by 75% while producing albumin-expressing cells at the end of stage 3 as shown in Figure 6A. We tested different ratios of dHCGM and stem cell differentiation media and found that stem cells did not survive and differentiate effectively in 100% dHCGM. A mixture of 25% stem cell and 75% hepatic maintenance media was found to be sufficient to drive differentiation.

In addition to immunofluorescence staining, we analyzed the expression of genes for endoderm, serum proteins produced by the liver, liver metabolic enzymes, drug transporters, and bile transporter proteins (see Figure 6B). Endoderm marker, SOX17 was significantly reduced, in microfluidic co-cultures compared to traditional 2D cultures. Gene expressions for A1AT and albumin, serum proteins produced by the liver, were significantly higher in microfluidic co-cultures compared to 2D culture, despite the fact that the former received 1/4 of the amount of the inductive cues (e.g., HGF and OSM) present in standard stem cell media (Figure 6C). In fact, gene expressions for liver metabolic enzymes (CYP3A4 and CYP2C9), drug transporters (OATP1B1 and OATP1B3), and bile transporter proteins (MRP2, MRP3, and MDR1) were significantly higher for stem cell-derived hepatocytes (SC-hepatocytes) differentiated in 3D microfluidic co-cultures compared to standard 2D cultures.

Is the presence of adult hepatocytes necessary to drive the hepatic differentiation of stem cells in a microfluidic device? To answer this question, we set up a microfluidic mono-culture experimental group and carried out hepatic differentiation of stem cells inside a microfluidic device in the absence of adult hepatocytes. The results of this experiment, shown in Figure S7, revealed that differentiating stem cells as microfluidic monocultures was less effective compared to co-cultures with adult hepatocytes. This is despite the fact that monocultures were differentiated in 100% stem cell media (stage 2 and 3 media), whereas co-cultures contained only 25% stem cell media. This experiment conclusively shows that adult hepatocytes are indeed important drivers of the differentiation process in microfluidic devices.

### 3.7. Benchmarking SC-Hepatocytes against Human Hepatocytes

**Figure 7:**
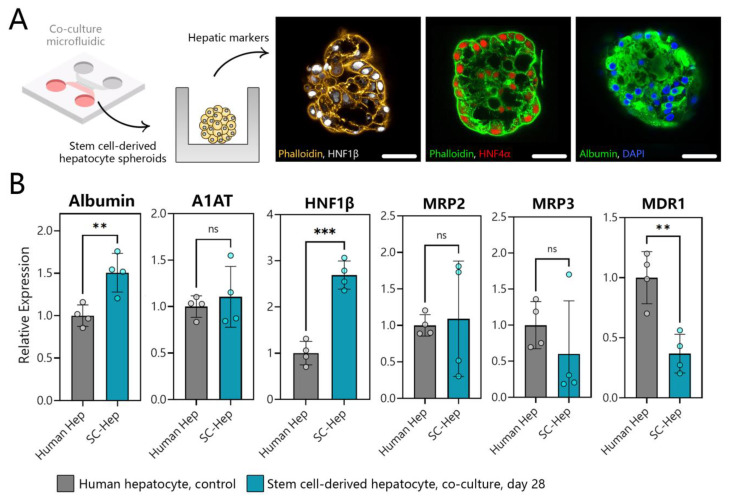
Assessing phenotype of SC-hepatocytes differentiated in microfluidic co-cultures. (**A**) Immunofluorescence staining for hepatic markers HNF-1β (white), HNF4α (red), and albumin (green) in SC-hepatocytes at day 21 in a microfluidic co-culture device (scale bar = 50 μm). (**B**) RT-PCR analysis of hepatic gene expression in human hepatocytes and SC-hepatocytes. ** *p* ≤ 0.01, *** *p* ≤ 0.001, ns (not significant).

After rigorously comparing hepatic differentiation in microfluidic co-cultures to a standard differentiation protocol, we proceeded to benchmark SC-hepatocytes against adult hepatocytes. We also showed that hepatic differentiation in microfluidic co-cultures results in higher differentiation than in microfluidic 3D monocultures (Figure S7). Immunofluorescence staining of SC-hepatocytes differentiated in microfluidic co-cultures showed high levels of the hepatic markers HNF4α, HNF1β, and albumin (Figure 7A). Albumin was expressed strongly and uniformly throughout an SC-hepatocyte spheroid in a manner resembling adult hepatocyte spheroids. HNF4α and 1β appeared as puncta in the nuclei at a frequency consistent with adult hepatocyte spheroids (see, for example, Figure 2D).

Analysis of hepatic gene expression in our SC-hepatocytes revealed a significant increase in albumin gene expression (~0.5 times) compared to human hepatocytes, a similar level of A1AT, and an increase in HNF-1β expression compared to human hepatocyte control. The gene expression for bile transporter proteins (MRP2 and MRP3) was also comparable between SC-hepatocytes and human hepatocytes, indicating successful differentiation (Figure 7B). While MDR1 expression in microfluidic SC-hepatocyte cultures was not as high as in human hepatocytes, it was significantly higher in than SC-hepatocytes produced in conventional 2D culture.

## 4. Discussion

In this study, we developed microfluidic 3D (spheroid) co-cultures to test the hypothesis that adult hepatocytes provide instructive cues to drive differentiation of stem cells along the hepatic lineage. We provide several novel observations. First, human hepatocytes isolated from mice with humanized liver cultured as spheroids in a microfluidic device remained highly functional and maintained a differentiated hepatic phenotype for at least 4 weeks. In fact, expression of several bile acid and drug transporters as well as CYP450 enzymes was higher at the end of the culture than at the start. While there have been previous examples of long-term human hepatocyte cultures, most notably by Khetani et al. [79], our cultures appear to be more functional in terms of albumin production and do not require supporting cells or micropatterned surfaces. Second, stem cells placed into microfluidic co-cultures benefit significantly from the presence of adult hepatocytes in the adjacent microfluidic compartment. Hepatic differentiation of stem cells is more pronounced in microfluidic co-cultures compared to a standard differentiation protocol. This is despite the fact that microfluidic co-cultures were exposed to 75% hepatic maintenance and 25% hepatic progenitor (S2) or hepatic maturation (S3) media, whereas standard cultures were in 100% stem cell media.

Why do microfluidic co-cultures enhance hepatic differentiation of stem cells? Our team has previously described enhanced production of HGF and other hepato-inductive signals in microfluidic cultures of adult hepatocytes. In the present study, we detected robust production of HGF in microfluidic spheroid cultures of human hepatocytes and modeled the distribution and accumulation of HGF in the microfluidic device. Our modeling predicts that HGF is concentrated in the source (hepatocyte) compartment, where it reaches 2 µg/mL, and from where it diffuses into the stem cell compartment, reaching a concentration of 0.5 µg/mL. We note that standard protocols for directed differentiation of hPSCs into hepatocytes use 10 to 20 ng/mL HGF and are likely limited by the rapid degradation of this morphogen. In our microfluidic device, HGF and other hepatocyte-secreted signals accumulate locally, in proximity to stem cells, and reach an order-of-magnitude higher concentration in the stem cell compartment. Unlike standard culture systems, where HGF and other inductive signals are added once per 24 or 48 h, these signals are secreted continuously by adult hepatocytes in the microfluidic device and are likely more bioactive.

## 5. Conclusions

In conclusion, this paper demonstrates that paracrine signals secreted by adult hepatocytes in microfluidic co-culture devices may be harnessed to drive differentiation of neighboring stem cells. We showed that hepatic differentiation of stem cells was more pronounced in microfluidic co-cultures compared to microfluidic monocultures or a standard hepatic differentiation protocol. Such co-cultures may minimize the need for expensive differentiation protocols relying on exogenous growth factors and may shed new light on the signals and mechanisms for hepatic differentiation.

## Data Availability

The datasets generated during and/or analyzed during the current study are available from the corresponding author on reasonable request.

## Supplementary Materials

The following supporting information can be downloaded at: https://www.mdpi.com/article/10.3390/cells12151982/s1, Figure S1: Process of fabricating microfluidic co-culture devices; Figure S2: Characterization of microwells for spheroid formation; Figure S3: Morphology and polarization of human hepatocyte spheroids in a microfluidic device; Figure S4: Modeling of hepatocyte growth factor (HGF) secretion in small- and large-volume cultures; Figure S5: Hepatic differentiation of stem cells using standard protocol; Figure S6: Expression of key markers during standard hepatic differentiation protocol; Figure S7: Differentiation of stem cells as mono-cultures in microfluidic devices; Table S1: Key resources table; Table S2: List of the primers; Table S3: List of the media and GFs.
