# Supplementary material for: Guiding Hepatic Differentiation of Pluripotent Stem Cells Using 3D Microfluidic Co-Cultures with Human Hepatocytes
Source: Cells. 2023 Aug 1;12(15):1982. doi: 10.3390/cells12151982 (PMC10417547; doi:10.3390/cells12151982)
Supplement: Supplementary file 1 [file cells-12-01982-s001.zip › cells-2503959-supplementary.pdf]

Supplemental data items

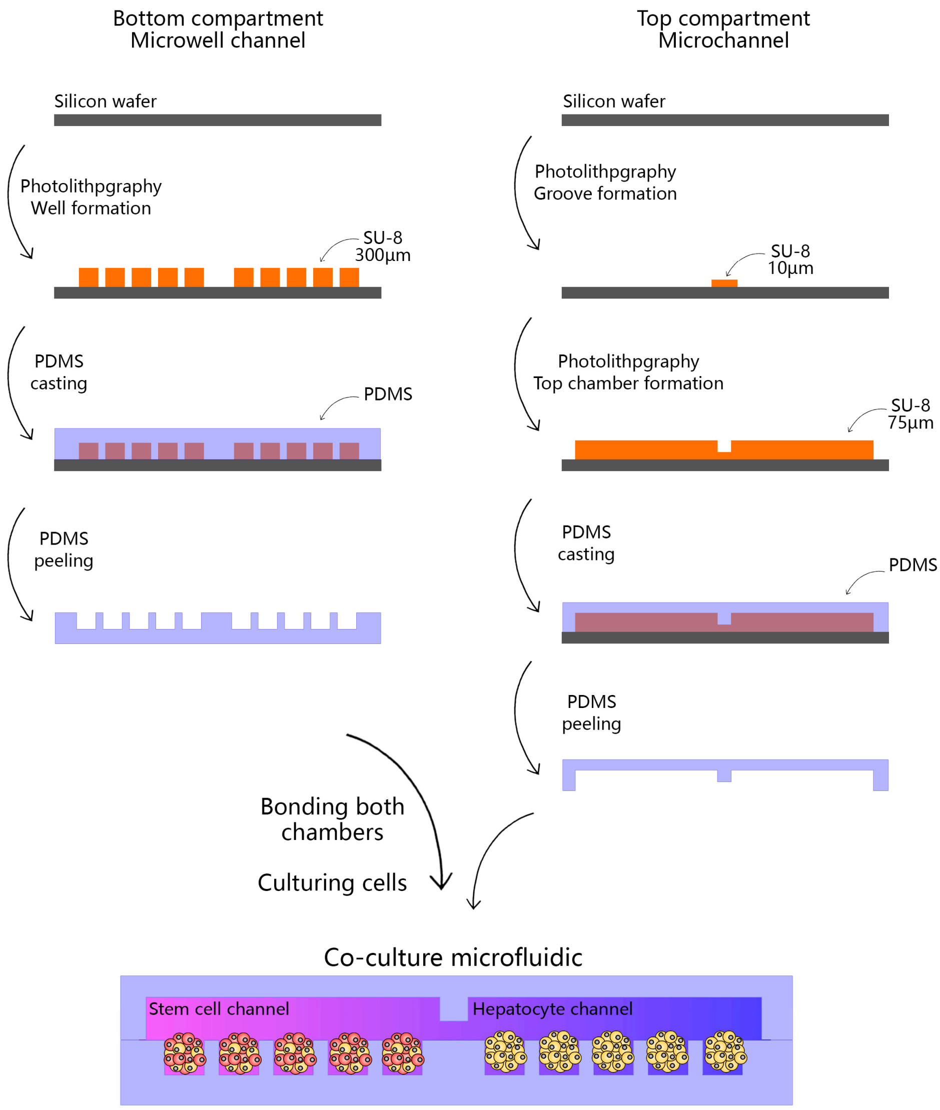

Figure S1. Process of fabricating microfluidic co-culture devices. Steps of the process to fabricate microwell and flow layers of the microfluidic device. Individual microwells were 300 µm diameter × 300 µm height. The flow layer contained fluidic channels with thickness of 75 µm. The master molds for each layer were fabricated using SU-8 photolithography and replicated in PDMS. After curing, the PDMS, the layers were peeled off and bonded together to create a microfluidic device.

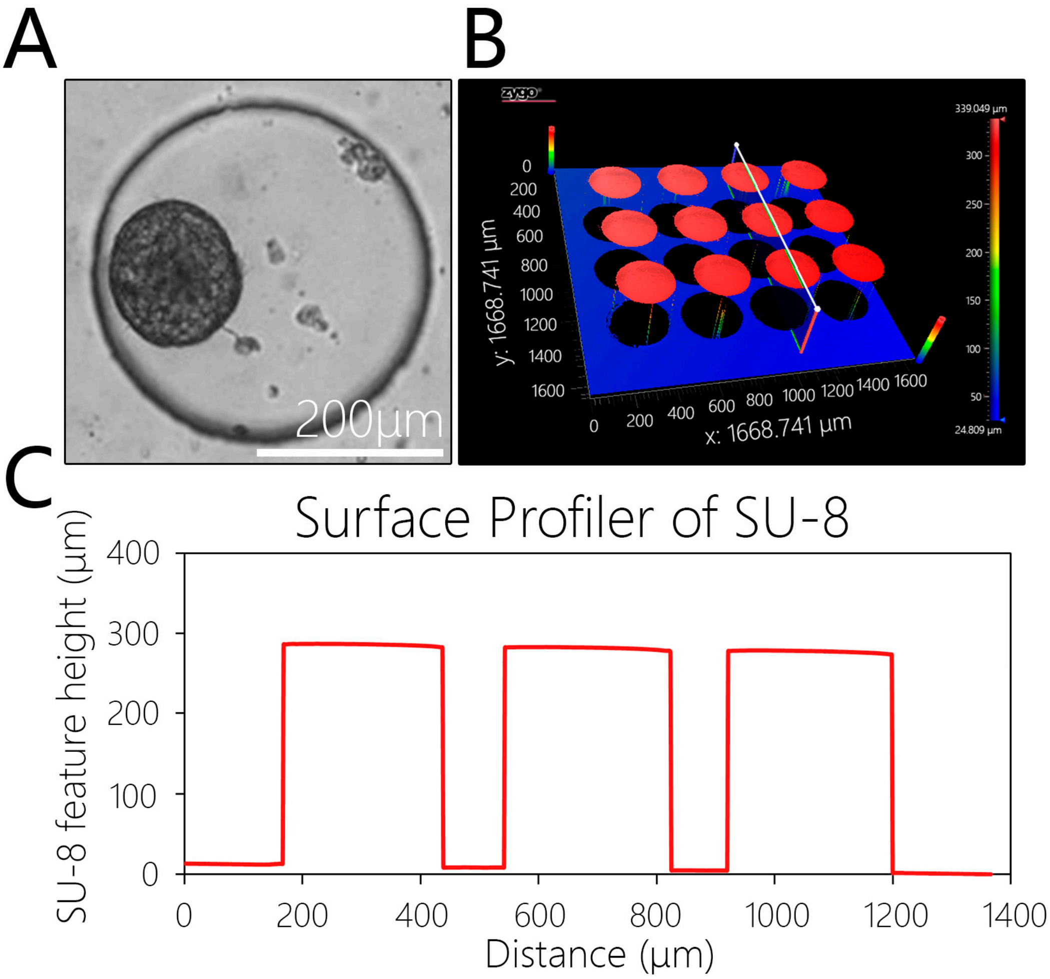

Figure S2. **Characterization of microwells for spheroid formation.** (A) Brightfield micrograph, showing a close-up image of a hepatocyte spheroid in a microwell. (B) Profilometry of the cell culture compartment, showing that wells were 300 µm deep. (C) Confirmation that features fabricated in SU-8 were consistent with dimensions of the wells.

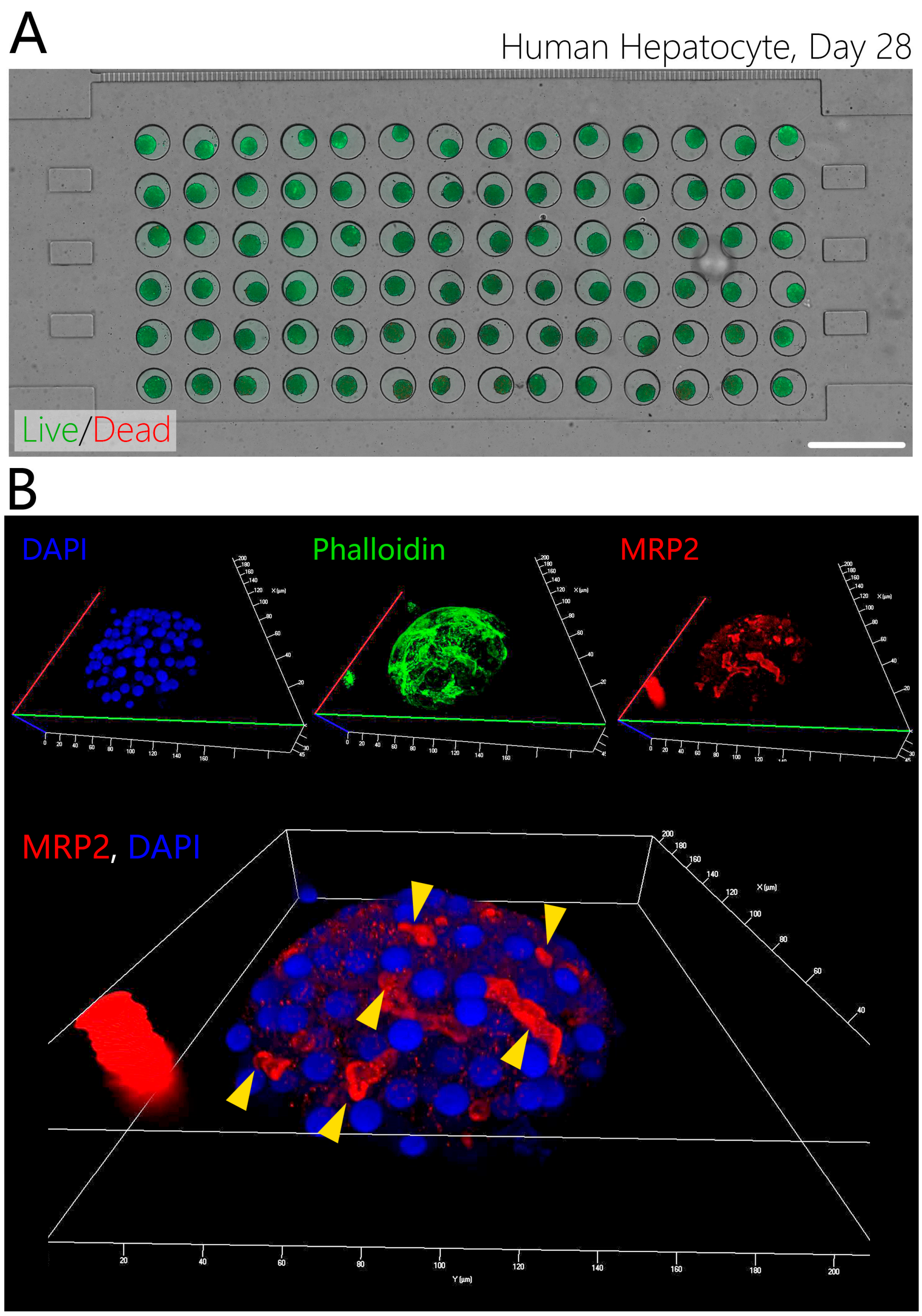

Figure S3. Morphology and polarization of human hepatocyte spheroids in a microfluidic device. (A) A zoomed-out view of an array of hepatocyte spheroids in a microfluidic device after 28 days of culture (scale bar = 1mm). Live/Dead staining reveals that cells are viable with minimal cell death observed. (B) A 3D reconstructed image of a hepatocyte spheroid stained for actin (green), MRP2 (red) and nuclei (blue, DAPI) to reveal bile canaliculi (highlighted using yellow arrows).

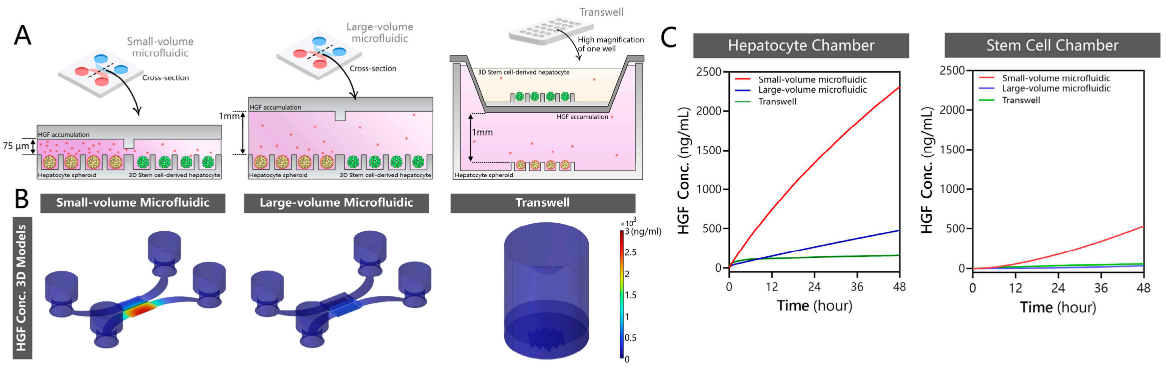

Figure S4. Modeling of hepatocyte growth factor (HGF) secretion in small- and large-volume cultures. (A) Schematic describing HGF production in three types of 3D cell culture systems: 1) Small-volume microfluidic, 2) large-volume microfluidic, and 3) transwell format. (B) COMSOL modeling heatmaps of hepatocyte growth factor (HGF) secretion in small-volume microfluidic, large-volume microfluidic and transwell at t=48h. (C) Graphs showing HGF accumulation in hepatocyte chamber (left graph) and stem cell chamber (right graph) over the course of 48 h in small- and large-volume cultures.

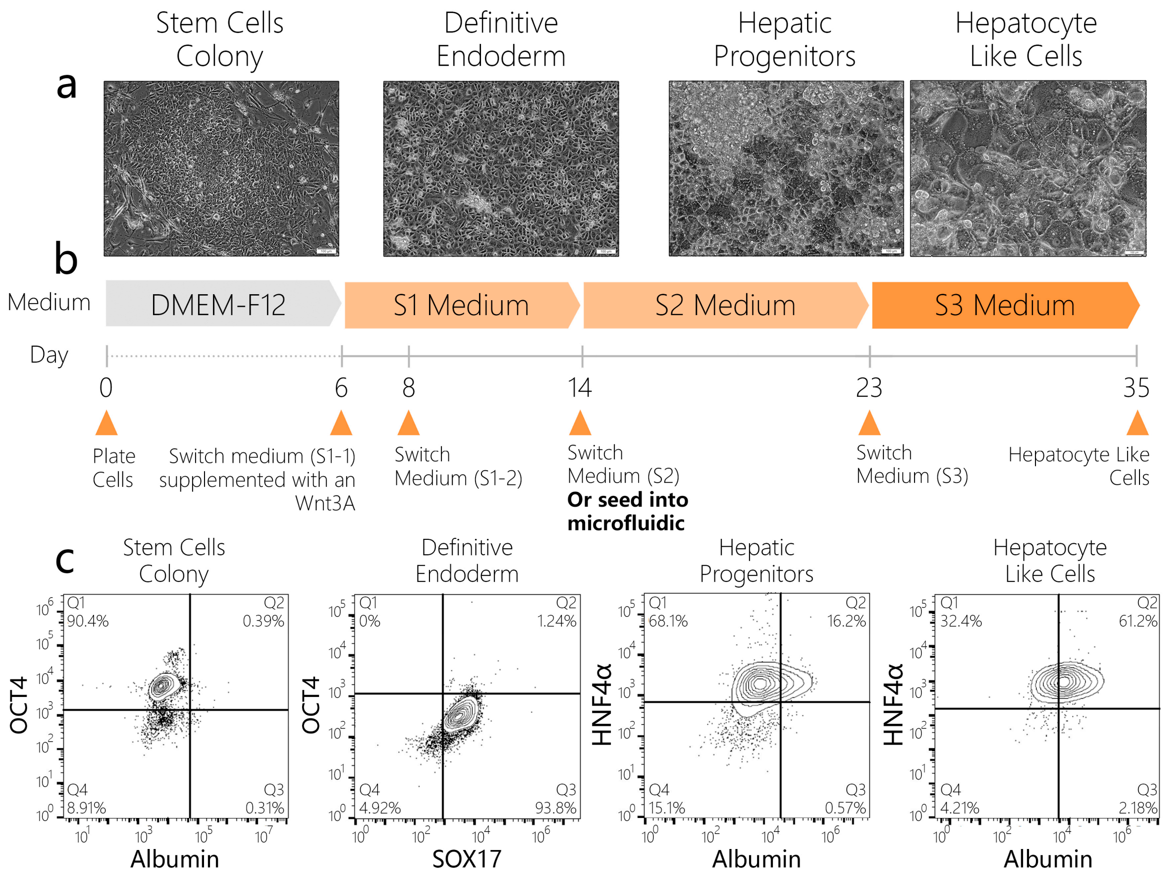

Figure S5. Hepatic differentiation of stem cells using standard protocol. (A) Changes in morphology of hESCs in the process of differentiation. (B) Workflow of the conventional 3-stage hepatic differentiation protocol. (C) Flow cytometry analysis at the end of each stage of the 35-day differentiation protocol. Cells from each stage were labeled for a stage-specific marker as follows - stage 0: OCT4+/albumin-, stage 1: OCT4-/SOX17+, stage 2: HNF4α+/albumin+, and stage 3: HNF4α+/albumin+.

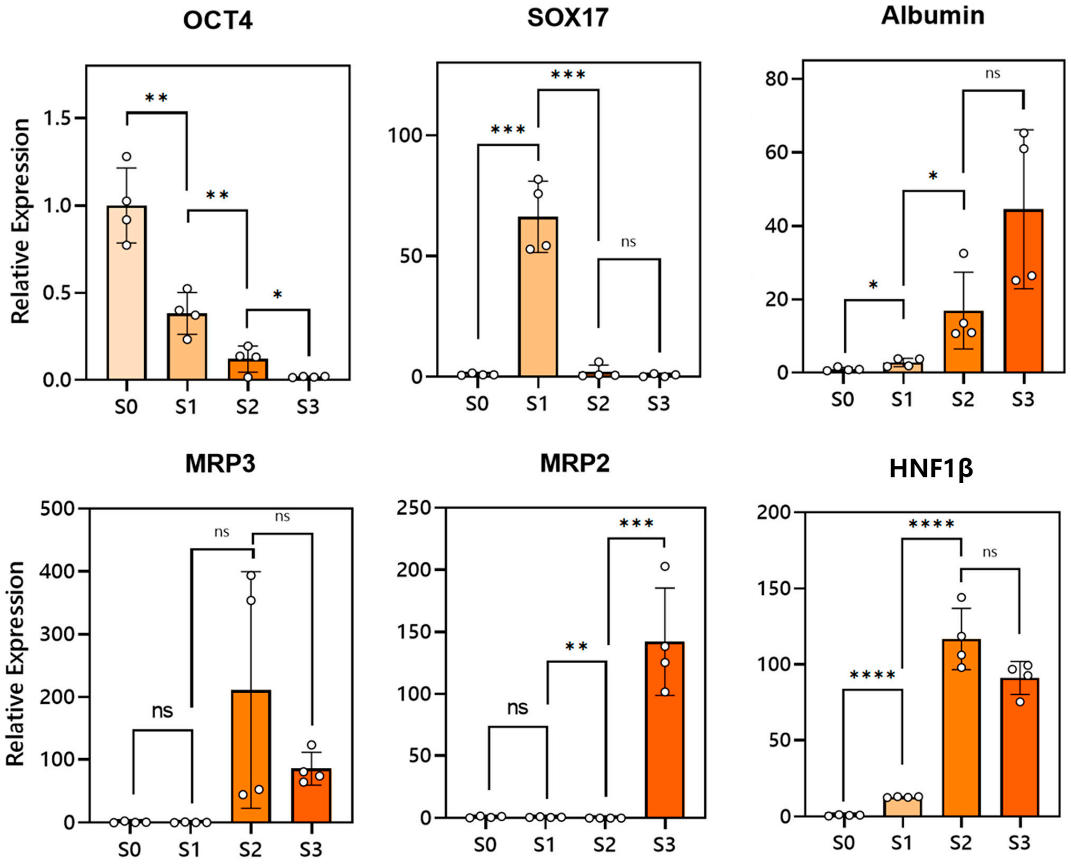

Figure S6. Expression of key markers during standard hepatic differentiation protocol. PCR-RT results for the differentiation of hepatocytes, showing stem cell marker OCT4, definitive endoderm SOX17, and hepatic markers albumin, MRP2, MRP3, HNF4β. These results show higher levels of hepatic marker expression at stage 3 (S3) of differentiation compared to other stages (Data are represented as mean ± SD, *$*P \leq 0.05$, **$P \leq 0.01$, ***$P \leq 0.001$, ****$P \leq 0.0001$*, n = 4).

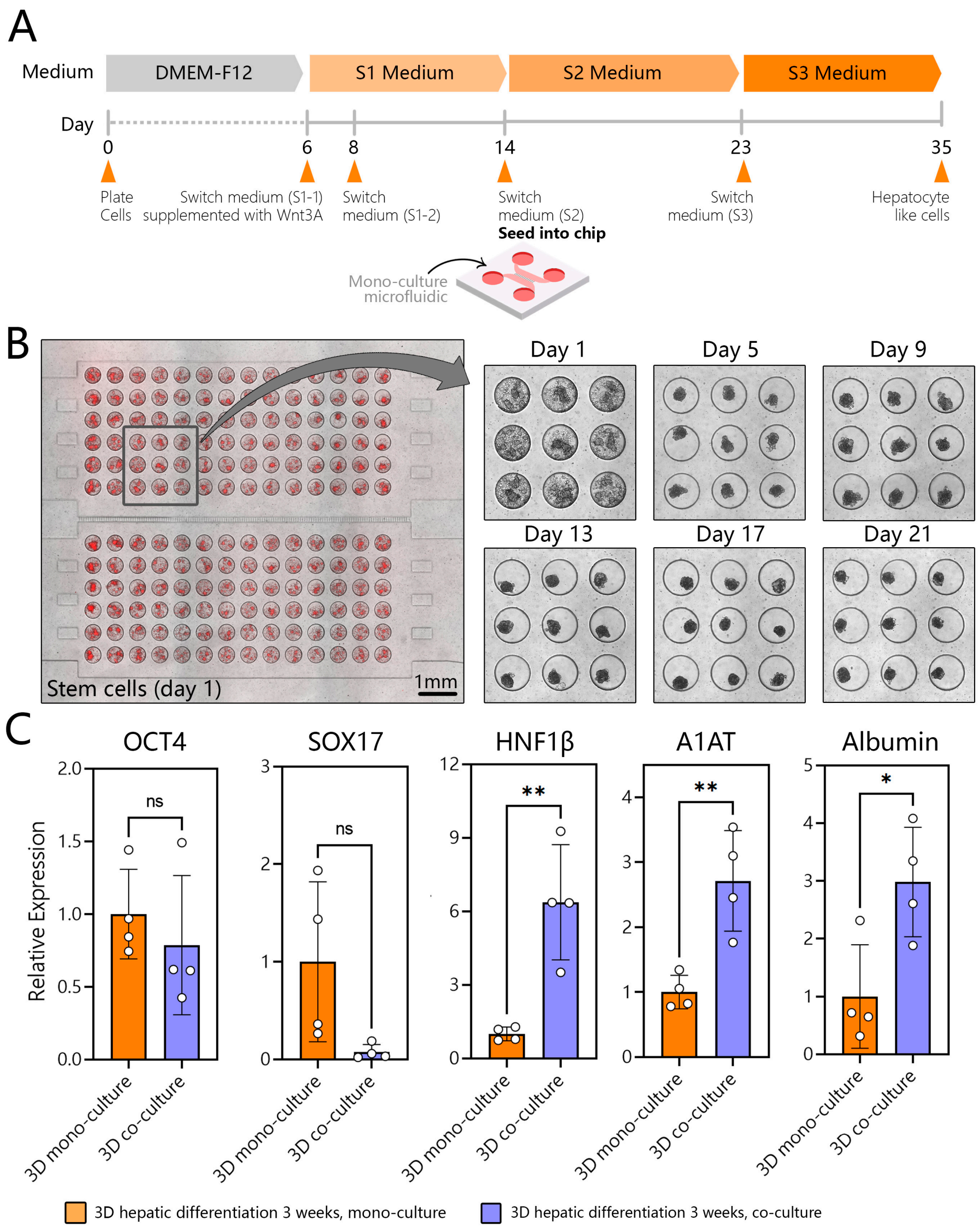

Figure S7. Differentiation of stem cells as mono-cultures in microfluidic devices. (A) Workflow for differentiating stem cell-like hepatocytes as a monoculture in a microfluidic device. (B) An image showing DE spheroids in a device as mono-culture. Note that stem cells contained mCherry (red fluorescence) reporter for SOX17 – marker of DE. Also note that the same DE cells were populating both compartments of the microfluidic device. The right panel shows DE spheroids at different timepoints during 3 weeks of culture. (C) RT-PCR analysis of stem cell marker (OCT4), DE marker (SOX17), hepatic markers (A1AT, albumin, HNF4β.). Data are presented as mean standard deviation, $*P \leq 0.05$, $**P \leq 0.01$.

Table S1. Key Resources table

Reagent or resource	Source	Identifier
**Antibodies**		
OCT4 (OCT3)	STEMCELL Technologies	Clone 3A2A20
SOX17	Santa Cruz	sc-17356
HNF4α	Santa Cruz	sc-8987
HNF1β	Santa Cruz	sc-22840
Albumin	Santa Cruz	sc-46293
AFP	Santa Cruz	sc-51506
MRP2	Santa Cruz	sc-5770
Alexa Fluor™ 647 Phalloidin	Thermofisher	A22287
Donkey anti-Goat Secondary Antibody Alexa Fluor™ 647	Invitrogen	A-21447
Donkey anti-Goat Secondary Antibody Alexa Fluor™ 546	Invitrogen	A-11056
Donkey anti-Mouse Secondary Antibody Alexa Fluor™ 546	Invitrogen	A-10036
Goat anti-Mouse Secondary Antibody Alexa Fluor™ 546	Invitrogen	A-11003
Goat anti-Rat Secondary Antibody Alexa Fluor™ 488	Invitrogen	A-11006
Donkey anti-Rabbit Secondary Antibody Alexa Fluor™ 594	Invitrogen	A-21207
**Chemicals, peptides, and recombinant proteins**		
KnockOut Serum Replacement	Gibco/Invitrogen	Catalog # 10828-028
2-Mercaptoethanol (55mM)	Gibco/Invitrogen	Catalog # 11140-050
Basic FGF (FGF2)	PeproTech	Catalog # 100-18B
B27 supplement	Life Tech	Catalog # 17504-044
Sodium Butyrate	Sigma	Catalog # B5887
Activin A	R&D Systems	Catalog # 338-AC
Wnt3a	R&D Systems	Catalog # 5036-WNP
Dexamethasone	Sigma	Catalog # D2915
Insulin	Novo Nordisk	Catalog # NDC 0169-1835-11
1-thioglycerol	Sigma	Catalog # M6145
HGF	R&D Systems	Catalog # 294-HGN
FGF4	R&D Systems	Catalog # 235-F4
BMP2	R&D Systems	Catalog # 355-BM
BMP4	R&D Systems	Catalog # 314-BP
DMSO	Sigma	Catalog # D2650
OSM	R&D Systems	Catalog # 295-OM-010/CF
**Critical commercial assays**		
Human Albumin ELISA Quantitation Set	Bethyl	E80-129

Experimental models: Cell lines		
Human Embryonic Stem cell line H9 RUNX1CGFP/w SOX17mCHERRY/w	MCRI Blood development Group	https://doi.org/10.1038/nbt.3702
Irradiated Mouse Embryonic Fibroblasts	R&D Systems	PSC001
Chimeric mice with humanized livers (PXB-mice®)	PhoenixBio Co. Japan	doi: 10.3390/ijms15010058
Oligonucleotides		
Primers for RT PCR	This paper	see Table S2

Table S2. List of the primers

Gene	Forward	Reverse
GAPDH	GGAGTCAACGGATTTGGT	AAGATGGTGATGGGATTTCCA
OCT4	GGAGTCAACGGATTTGGT	AAGATGGTGATGGGATTTCCA
SOX17	GGAGTCAACGGATTTGGT	AAGATGGTGATGGGATTTCCA
Albumin	GCACAGAATCCTTGGTGAACAG	ATGGAAGGTGAATGTTTTCAGCA
A1AT	ACTGTCAACTTCGGGGACAC	CATGCCTAAACGCTTCATCA
HNF1β	GTACGTCAGAAAGCAACGAGAGAT	TGACTGCTTTTGTCTGTCATATTTCCA
BSEP	AAATATGCTTTTGGGTCATTG	GTCAGCTATGGCATCATTG
MRP2	TCCAACTGTGCTTCAAGC	GGCATCCACAGACATCAG
MRP3	CCTGCTGATACAGTATGAGCGGC	TGTAGAAGGTGGTGAAGCGGAAG
MDR1	GTCATCGCTGGTTTCGATGATG	ATTTCCTGCTGTCTGCATTGTG
CYP1A2	GCTTCTACATCCCCAAGAAAT	ACCACTTGGCCAGGACT
CYP2C9	CCAGATCTGCAATAATTTTTCTC	CAAGCTTTCAATAGTAAATTCAGATG
CYP2D6	CTTGGACAAAGCCGTGA	GACAGCATTCAGCACCTC
CYP3A4	ACTGCCTTTTTTGGGAAATA	GGCTGTTGACCATCATAAAAG
OATP1B1	TCATACTCTGTGAAAACAAATCAG	CAGACTGGTTCCCATTGAC
OATP1B3	CTCTGTTTGCTAAAATGTACGTG	GAAGAAATAATGGAAAATAGTCCAG

Table S3. List of the media and GFs

	dHCGM	S2 media	S3 media
Basal media	DMEM	IMDM	IMDM
Glucose	1g/L	4.5g.L	4.5g.L
NaHCO3	3.7g/L	36mM	36mM
HEPES	w/0	25mM	25mM
Sodium pyruvate	0.11g/L	1mM	1mM
HEPES	20mM		
Penicillin/Strep	100IU/mL	1%	1%
FBS	10%	20%	5%
Insulin (Novolin)	0.25ug/mL	0.126U/ml	0.126U/ml
Dexamethasone	50nM	100 nM	100 nM
EGF	5ng/mL		
L-Proline	15ug/mL		
L-ascorbic Acid 2P	0.1mM		
DMSO	2%	0.50%	0.50%
1-thioglycerol		0.3mM	
HGF		20 ng/ml	20 ng/ml
FGF4		20 ng/ml	20 ng/ml
BMP2		10 ng/ml	
BMP4		10 ng/ml	
OSM			10 ng/ml
